# Uniportal thoracoscopic bullectomy with improved parietal pleurectomy for primary spontaneous pneumothorax

**DOI:** 10.1111/crj.13722

**Published:** 2023-12-03

**Authors:** Ningning Kang, Hao Zheng, Wei Ge, Jin‐Xiu Hu, Wen Liu, Ren‐Quan Zhang

**Affiliations:** ^1^ Department of Thoracic Surgery First Affiliated Hospital of Anhui Medical University Hefei China

**Keywords:** parietal pleurectomy with bullectomy, preserved parietal pleura, primary spontaneous pneumothorax, recurrence, thoracoscopic, uniportal

## Abstract

**Introduction:**

Parietal pleurectomy with bullectomy has been established as an effective method for preventing the recurrence of primary spontaneous pneumothorax (PSP). Our center introduced enhanced technical measures in uniportal thoracoscopic parietal pleurectomy with bullectomy for patients with PSP, aiming to document our initial experience and assess the procedure's effectiveness in preventing the recurrence of PSP.

**Methods:**

We analyzed the clinical data of 86 patients with PSP who underwent the improved uniportal thoracoscopic parietal pleurectomy with bullectomy between July 2019 and August 2022. During the procedure, the parietal pleura above the second intercostal space was stripped but not removed. Instead, it was retained in the thoracic cavity using a piece of pedunculated pleura. Subsequently, the stumps of the lung were covered by the preserved parietal pleura.

**Results:**

The results of the study showed that the mean operative time was 59.87 ± 16.93 min, and the postoperative drainage duration averaged 3.94 ± 2.17 days. The mean intraoperative blood loss was 24.33 ± 48.91 ml, and the mean postoperative drainage volume was 289.00 ± 170.03 ml. Prolonged air leakage for more than 5 days was observed in five patients (5.81%), but no other postoperative complications were recorded. During the follow‐up, one patient (1.16%) experienced a recurrence of pneumothorax.

**Conclusions:**

The perioperative results of bullectomy with the improved pleurectomy technique are deemed satisfactory. The various technical steps attempted at our center are found to be feasible and safe, and they may contribute to reducing the rates of recurrence in PSP.

## Graphical abstract

The study suggests that the improved uniportal thoracoscopic bullectomy with parietal pleurectomy technique had satisfactory perioperative results, which was safe and potentially effective in reducing recurrence rates of PSP.

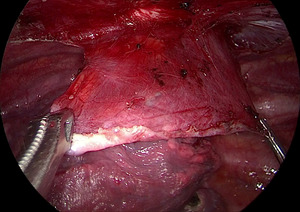

## 1 INTRODUCTION

Primary spontaneous pneumothorax (PSP) is a common emergency in thoracic surgery that occurs in patients without any apparent underlying lung disease. Various treatment options are available for PSP, including bed rest, closed chest drainage, and thoracoscopic‐assisted bullectomy.^1^ The American College of Chest Physicians (ACCP) and British Society of Thoracic Surgeons (BTS) guidelines state that surgery should be the next option if the air leak continues for more than 4 days.^2^, ^3^ The uniportal thoracoscopic approach has been demonstrated to be a less invasive technique and is associated with better cosmetic satisfaction.^4^, ^5^

Parietal pleurectomy is a surgical procedure that involves the removal or stripping of the parietal pleura, which is the outer layer of the membrane that surrounds the lungs. It is often performed as part of the treatment for PSP, a condition characterized by the sudden collapse of a lung without any apparent cause.^6^ Research and clinical evidence have shown that parietal pleurectomy can be an effective method to prevent the recurrence of PSP. By removing or stripping the parietal pleura, the surgical procedure aims to eliminate the potential spaces where air can accumulate, leading to a collapsed lung. This helps to decrease the likelihood of future episodes of pneumothorax.^7^

Parietal pleurectomy is typically performed using minimally invasive techniques, such as video‐assisted thoracoscopic surgery (VATS), which involves making small incisions and using a tiny camera and specialized instruments to visualize and operate on the pleural cavity. Comparing this method to conventional open surgery, reduced postoperative discomfort, shorter hospital stays, and quicker recovery are the outcomes.^8^ However, it is important to note that the effectiveness of parietal pleurectomy may vary depending on various factors, including the underlying cause of PSP, the extent of lung damage, and individual patient characteristics. Additionally, like any surgical procedure, parietal pleurectomy carries potential risks and complications, which should be carefully considered and discussed with a qualified healthcare professional before making treatment decisions.^9^

However, there is limited literature available on uniportal thoracoscopic parietal pleurectomy with bullectomy for PSP, and the reported procedures still carry a certain risk of recurrence. In 2019, our center started implementing some improved technical steps in uniportal thoracoscopic parietal pleurectomy with bullectomy for PSP patients. Instead of removing the parietal pleura, it was retained in the thoracic cavity using a piece of pedunculated pleura. In this study, we present our preliminary experience with 86 cases to initially evaluate the effectiveness and safety of this technique of operation.

## 2 METHODS

### 2.1 Ethics statement

This retrospective study was approved by the ethics committee of First Affiliated Hospital of Anhui Medical University, Hefei, China (Approval number: Quick PJ 2023‐01‐21).

### 2.2 Patients and clinical data

In this study, we included the clinical data of 86 patients with PSP who underwent uniportal thoracoscopic improved parietal pleurectomy with bullectomy between July 2019 and August 2022. Inclusion criteria were as follows: (1) Patients were diagnosed with PSP based on their symptoms and chest computed tomography. (2) Patients ranged in age from 12 to 60 years. (3) Cases included patients who relapsed after conservative treatment or continued to leak for more than 7 days after conservative treatment. (4) All cases of pneumothorax were primary and occurred spontaneously. (5) Patients underwent uniportal thoracoscopic improved parietal pleurectomy with bullectomy. Exclusion criteria were as follows: (1) Patients had a history of chest trauma, infectious diseases (such as pneumonia or tuberculosis), previous thoracic surgery, chronic lung diseases (asthma, pulmonary fibrosis, such as chronic obstructive pulmonary disease, or pneumoconiosis), or other systemic diseases. (2) The uniportal procedure converted to a multiportal or open operation.

### 2.3 Surgical procedure

**FIGURE 1:**
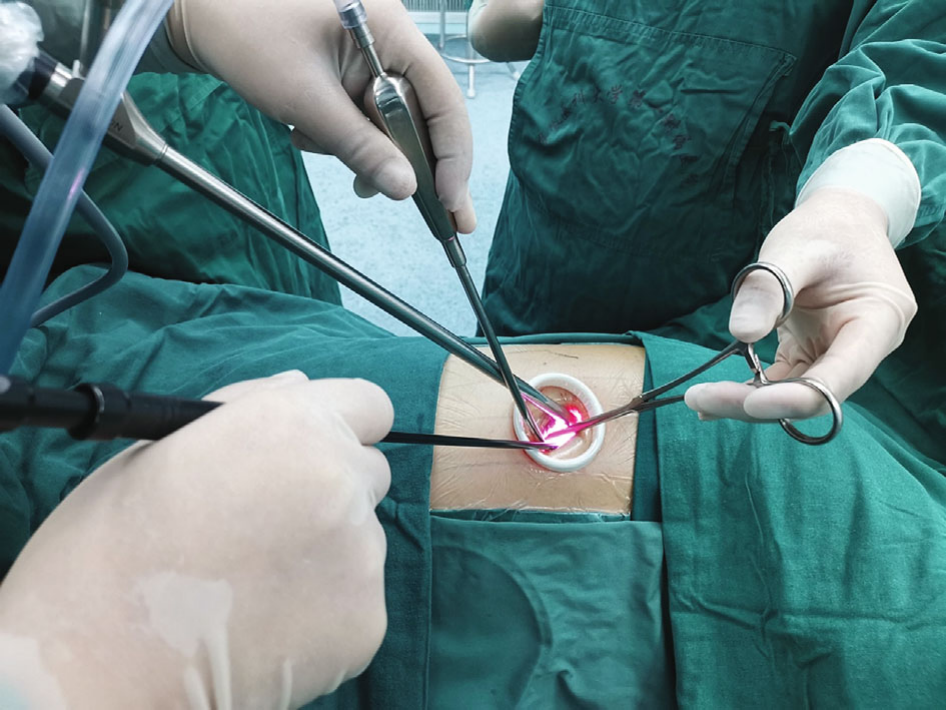
The thoracoscope was inserted into the thorax after a wound retractor was placed.

**FIGURE 2:**
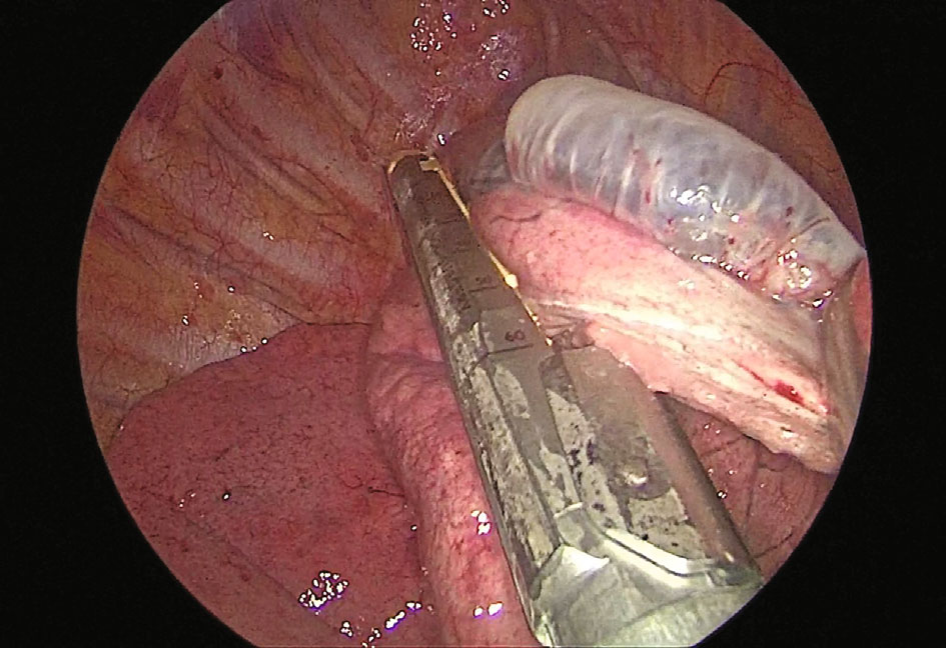
The blebs were resected using a linear stapler through the single incision.

The patients underwent general anesthesia with double‐lumen tubes and were positioned laterally. The operating surgeon stood on the patient's ventral side while the assistant stood behind the patient. A 2.5‐cm incision was made in the fourth intercostal space along the posterior axillary line, and an incision protector (wound retractor) was placed. A 10‐mm 30° thoracoscope was inserted into the thorax to identify the area of leakage after deflating the lung on the operative side (Figure 1). Blebs or bullae were lifted using an oval forceps and resected with a linear stapler through the single incision, which was carried out in all patients (Figure 2).

**FIGURE 3:**
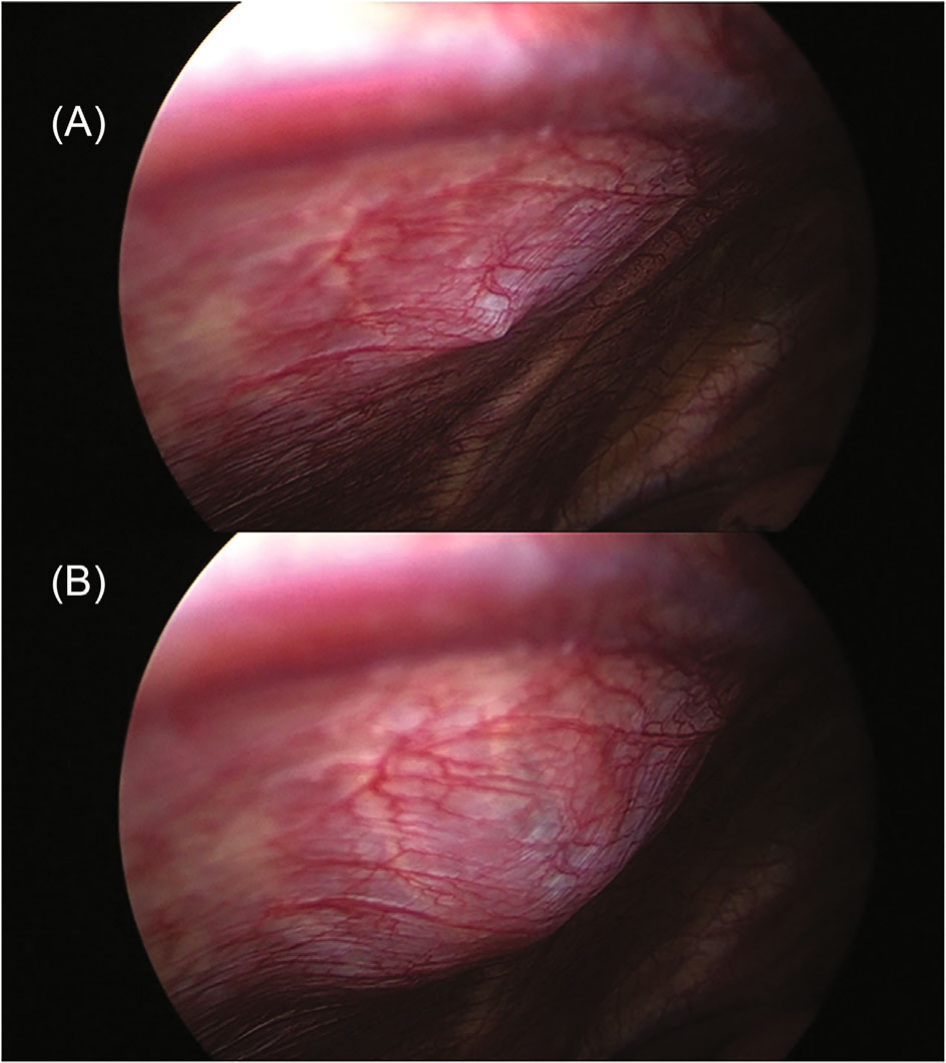
Normal saline was injected to separate the pleura from the chest wall muscle. (A) The tip of the syringe was inserted into the subpleura. (B) Normal saline was injected.

**FIGURE 4:**
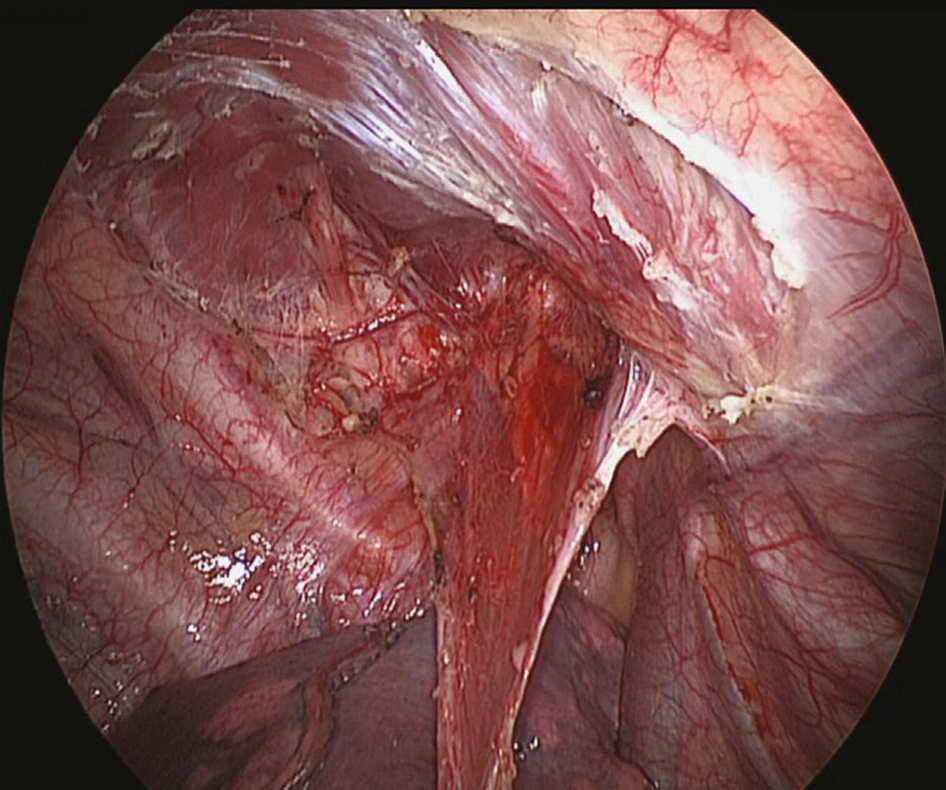
Dissection was carried out up to the edge of the sympathetic chain and outside of the internal mammary artery.

**FIGURE 5:**
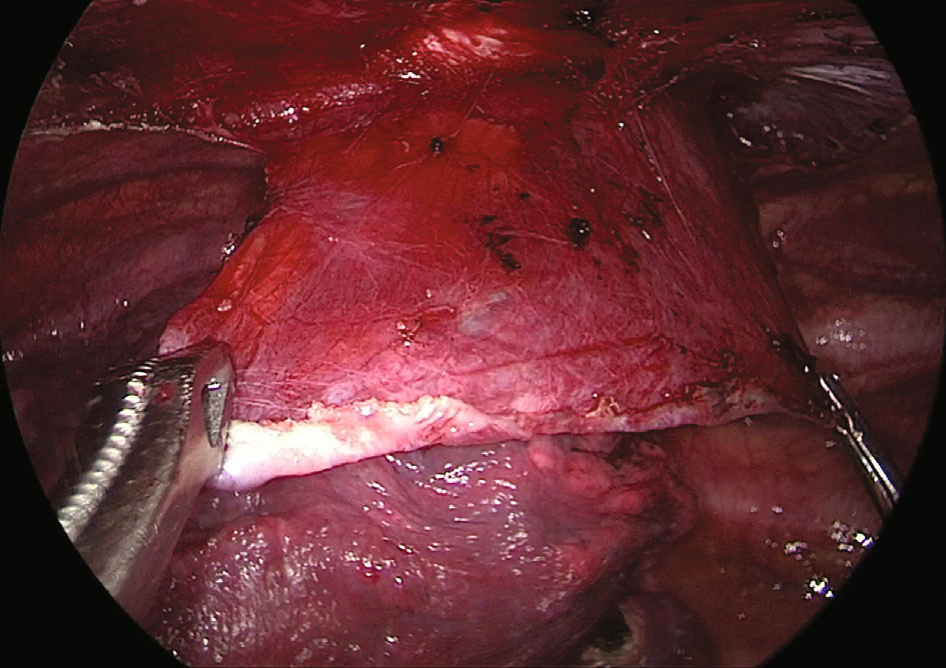
The stumps of the lung were covered by the resected parietal, which was not removed but retained in the thoracic cavity through a pedunculated pleura.

**FIGURE 6:**
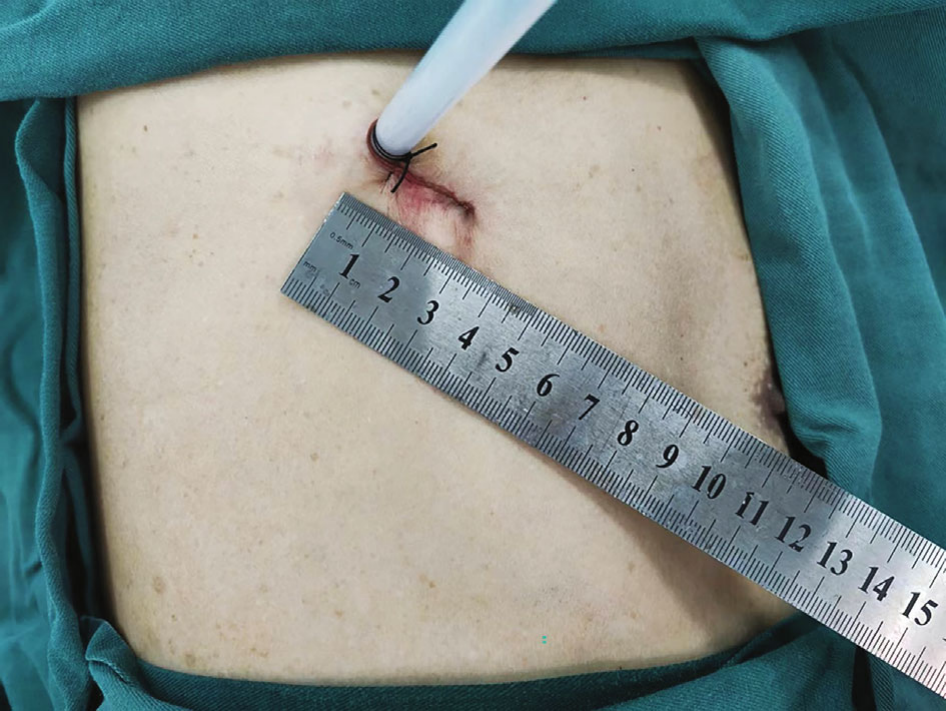
The tiny incision was made in the fourth intercostal space along the posterior axillary line.

To facilitate pleural dissection, a 5‐ml syringe filled with normal saline was inserted into the parietal pleura from the skin through the second intercostal space in the midaxillary line. The saline injection created a localized separation between the pleura and the chest wall muscle (Figure 3). The parietal pleura was then bluntly separated using a pair of scissors and aspirators. The dissection was performed up to 1 cm to the edge of the sympathetic chain and outside of the internal mammary artery (Figure 4). The resected pleura was not removed but instead retained in the thoracic cavity through a pedunculated pleura (Figure 5). Following air detection and hemostasis, a 28 French (Fr) chest drainage tube was inserted through the incision at the apex of the thoracic cavity. The stumps of the lung were covered by the preserved parietal pleura using an oval forceps. After the lung re‐inflated just as it was approaching the chest wall, the oval forceps was removed. So the pleura did not malposition. Finally, the incision was closed layer by layer after lung inflation (Figure 6).

Following the operation, all patients were provided a normal diet and received preventive antibiotics for 48 h. Chest radiographs were performed on the first day after the procedure. If no pleural effusion or pneumothorax was detected, and there were no signs of fever, hemoptysis or abnormal drainage, the drainage tube was removed. Subsequently, the patients were discharged.

All discharged patients were followed up by regular outpatient review and telephone return visits, with the longest follow‐up being 36 months and the shortest follow‐up being 12 months. During the follow‐up period, recurrence of pneumothorax was observed in one patient. Recurrence was defined as the development of pneumothorax on the same side that was operated on, occurring more than 1 month after the patient's discharge.

## 3 RESULTS

### 3.1 Patient demographics and clinical profile

Among the 86 patients included in the study, three individuals encountered prolonged air leakage lasting more than 5 days and initially received closed chest drainage as a treatment approach. Subsequently, they were transferred to our department from the Respiratory Medicine Department for further treatment. The clinical and pathological characteristics of these patients, along with other relevant information, are presented in Table 1. The study cohort consisted of 72 (83.72%) men and 14 (16.28%) women, with a median age of 20 years (range 15–59). Among the patients, 8 (9.30%) had a history of smoking. The mean body mass index (BMI) was 19.23 ± 2.20. All surgical procedures were performed by the same surgical team. The performance status of the patients is also provided in Table 1.

**TABLE 1:** Patient characteristics.

Variable	No. of cases (%), *n* = 86
Sex	
Male	72 (83.72)
Female	14 (16.28)
Age	
Mean ± *SD*	26.08 ± 12.90
Median (range)	20 (15–59)
Laterality	
Right	40 (46.51)
Left	46 (53.49)
Both	0
BMI	
Mean ± *SD*	19.23 ± 2.20
Median (range)	18.62 (15.19–22.84)
Smoking history	8 (9.30)
Performance status (ECOG)	
0	86 (100)
1	0

Abbreviation: BMI, body mass index.

### 3.2 Operative data analysis of patients undergoing enhanced uniportal thoracoscopic parietal pleurectomy with bullectomy for PSP

The surgical data are summarized in Table 2. The mean operative time for the uniportal thoracoscopic parietal pleurectomy with bullectomy was 59.87 ± 16.93 min. The postoperative drainage duration averaged 3.94 ± 2.17 days. None of the cases required conversion to two‐ or three‐port VATS during the operation. Open surgery was not performed on any patient, and no blood transfusions were administered. The mean intraoperative blood loss was 24.33 ± 48.91 ml, and the mean postoperative drainage volume was 289.00 ± 170.03 ml. Prolonged air leakage for more than 5 days occurred in five patients (5.81%).

**TABLE 2:** Operative data for 86 patients with primary spontaneous pneumothorax who underwent improved uniportal thoracoscopic parietal pleurectomy with bullectomy.

Operative data	
Operative time (min), mean ± *SD*	59.87 ± 16.93
Perioperative blood loss (ml), mean ± *SD*	24.33 ± 48.91
Postoperative drainage (ml), mean ± *SD* Open conversion, *n* (%)	289.00 ± 170.03 0
Postoperative hospital stay (days), mean ± *SD*	4.88 ± 2.16
Chest drainage time (days), mean ± *SD*	3.94 ± 2.17
Prolonged air leakage (>5 days), *n* (%)	5 (5.81%)
Recurrence, *n* (%) Reoperation, *n* (%)	1 (1.16%) 0

Postoperative pathology confirmed the presence of blebs/bullae in all cases. No patients required open conversion or reoperation, and no other complications were recorded.

The recurrence of pneumothorax occurred in only one patient (1.16%) after 14 months following discharge. Upon reviewing the patient's chest CT, it was observed that new blebs/bullae had appeared near the stumps of the lung. This recurrence may have been caused by the emergence of these new blebs/bullae. The patient was treated conservatively and discharged after 4 days.

## 4 DISCUSSION

A recurrence may not necessarily subsequent of suture leakage but mainly from new dystrophic areas, especially in the patients with specific pathologies and/or that not stop smoking. PSP occurs when there is a rupture of subpleural blebs (smaller than 2 cm) or bullae (larger than or equal to 2 cm) with the presence of air in the pleural space, leading to lung collapse. The recurrence rate for patients treated conservatively alone is approximately 32%.^10^ VATS bullectomy is considered the gold standard treatment for controlling pneumothorax in patients with PSP.^11^ However, recurrence rates following bullectomy alone remain relatively high. In a randomized controlled trial reported by Min et al. in 2014,^12^ the recurrence rate after bullectomy alone was 7.5%.

Currently, there is no consensus on the optimal surgical approach to minimize the risk of pneumothorax recurrence. Pleurectomy, which involves the removal of the parietal pleura, is often performed in conjunction with bullectomy for patients considered to be at high risk of recurrence.^13^ Several studies have shown that pleurectomy is more effective than pleural abrasion in controlling pneumothorax recurrence, despite being a more complex surgical procedure.^14^, ^15^ In 2001, Migliore et al.^16^ first reported the use of uniportal VATS in thoracic surgery. With advancements in uniportal endoscopic surgery and instruments, some surgeons have attempted parietal pleurectomy for PSP using more minimally invasive approaches compared to the traditional three‐port or two‐port techniques. However, partial pleurectomy also makes subsequent access for other pathologies more difficult.^15^

In this study, the uniportal approach was utilized, where both the thoracoscope and instruments were inserted through a single incision into the pleural cavity simultaneously. However, this simultaneous insertion may cause interference between the instruments and the thoracoscope, especially for surgeons who are new to this technique. Additionally, as the pleura begins to separate from the chest wall after bullectomy, it becomes more challenging to perform the procedure due to the smaller angle between the instruments and the chest wall.^17^, ^18^

To address these challenges in this study, a technique was employed in which 5 ml of normal saline was injected into the latent gap between the pleura and the chest wall muscle. This injection served two purposes: It increased the angle between the instruments and the chest wall and loosened the parietal pleura, making it easier to dissect. Although this additional step increased the operative time, it reduced the difficulty of parietal pleura dissection and facilitated subsequent dissections, resulting in convenient and fast procedures.

The mean operating time in this study was 59.87 ± 16.93 min, which is comparable with the results reported by Ocakcioglu et al.,^19^ where the mean operating time was 58.40 ± 6.92 min. Ryohei et al.^20^ suggested that for uniportal pleurectomy, an incision can be made in the fifth or sixth intercostal space on the anterior axillary line. PSP often occurs in taller individuals with a narrow and elongated chest cavity. According to the recommendations of Ryohei et al.,^20^ choosing the fifth or sixth intercostal space for the incision may result in the surgical instruments being unable to reach the apex of the chest due to the longer distance. This would make it impossible to complete the detachment of the apex pleura. However, using the fourth intercostal space avoids this issue, making the procedure more comfortable and smooth to perform. Therefore, it has a broader applicability. In this study, the fourth intercostal space along the posterior axillary line was chosen as the incision site. This decision was made to shorten the operating distance, increase the operating angle and accommodate patients with taller physiques. Furthermore, the incision location on the posterior axillary line ensured that it would be hidden by the patient's arm after the operation, improving cosmetic outcomes.

Prolonged air leakage after surgery and recurrence are common complications that surgeons aim to prevent in the surgical treatment of PSP. Parietal pleurectomy has been recognized as an effective method to reduce the incidence of recurrence, as demonstrated in various studies. In a study by Brophy et al.,^13^ it was reported that the recurrence rate was significantly lower in patients who underwent both bullectomy and pleurectomy compared to those who underwent bullectomy alone. The recurrence rate for patients who underwent bullectomy and pleurectomy was 5.9%, while it was 15.1% for patients who underwent bullectomy alone. This finding highlights the beneficial impact of pleurectomy in reducing the risk of recurrence. The combination of bullectomy and pleurectomy has been shown to provide improved outcomes in terms of preventing recurrence. Pleurectomy involves the removal or stripping of the parietal pleura, which helps to create an adhesive environment between the lung and chest wall, reducing the potential space for air accumulation and preventing recurrence of pneumothorax. By combining bullectomy (removal of subpleural blebs or bullae) with pleurectomy, surgeons can address both the source of air leakage and minimize the risk of recurrence. This combined approach aims to provide better long‐term outcomes for patients with PSP undergoing surgical treatment.^15^, ^21^

Prolonged air leakage was observed in five patients (5.81%) during the study. Several risk factors may contribute to air leakage—(1) tiny blebs: three patients had numerous tiny blebs in their lung tissue, aside from the ruptured blebs/bullae; (2) lung blebs below the lung surface: these blebs may not have been completely sealed when the tissues were stapled during the procedure. Consequently, leakage was observed during the washing of the pleural cavities. However, after suturing the stumps using 3‐0 Prolene, the leakage decreased and eventually disappeared within 6–10 days; (3) parietal pleura broken: in the other two patients, the parietal pleura was found to be broken during separation, indicating insufficient coverage of the stumps. It is believed that the preserved parietal pleura can help reduce the recurrence rates. By covering the stumps of the lung with the preserved parietal pleura, better adhesion between the lung tissue and chest wall can be promoted. The dissected pleura was connected to the chest wall through a piece of pedunculated tissue to maintain the capillary blood supply to the pleura. While special materials like Neoveil or biomedical fibrin glue may be available to prevent stump leakage, their high cost and limited availability in less developed areas or countries may hinder their widespread use.^22^, ^23^ Therefore, the technique of preserving the parietal pleura offers a practical and accessible method to enhance outcomes and reduce recurrence rates in such settings.

In this study, the mean perioperative blood loss was 24.33 ± 48.91 ml. However, one patient experienced intercostal artery bleeding during the operation, which contributed to the higher blood loss. The bleeding was not promptly controlled due to the small incision size, which made exposure challenging. Concerns have been raised by some surgeons regarding the increased risk of hemothorax after pleurectomy, as the parietal pleura contains abundant capillaries. Dżeljilji et al.^9^ reported a hemothorax rate of 12.32% in patients who underwent parietal pleurectomy. In their study, five patients were treated with puncture and the remaining four required access via thoracotomy. It is important to note that the range of parietal pleurectomy performed in their study was extensive, from the top to the diaphragm, which differs from the approach used in this study. Fourteen patients who underwent parietal pleurectomy saw an average intraoperative blood loss of 92.9 ± 37.1 ml, according to Cai et al.^7^ During their process, the parietal pleura's dissection edge stretched from the first rib to the level of the diaphragm, in front to the side of the sternum, and in the back to about 1 cm from the sympathetic chain. This extensive pleurectomy resulted in a larger drainage volume (510.7 ± 269.4 ml). Based on the findings of this current study, the authors suggest that stripping the parietal pleura above the second intercostal space is sufficient. The dissection edge should not involve the mediastinal pleura, and it should be located anterior to 1 cm from the internal mammary artery and posterior to 1 cm from the sympathetic chain. Additionally, after completing the parietal pleurectomy, the authors used gauze or cotton pads to compress the wound for 5 to 10 min to control capillary bleeding effectively.

The study has several limitations. First, the number of cases included in the study was limited, which may affect the generalizability of the findings. Additionally, this was a retrospective study, which carries inherent limitations such as potential biases and confounding factors. Another limitation is that the study did not compare the uniportal VATS treatment of spontaneous pneumothorax with other surgical methods such as multiport VATS or uniportal thoracoscopic bullectomy with pleural abrasion. A comparative analysis could provide further insights into the effectiveness and safety of different surgical approaches. Furthermore, the lack of long‐term follow‐up data raises uncertainty about the occurrence of pneumothorax recurrence beyond the 30‐month period. Longer term follow‐up would be valuable in assessing the durability of the surgical outcomes. Lastly, the study acknowledges the need for randomized controlled trials to confirm the effectiveness of the technical maneuvers employed in this study. Randomized controlled trials can provide higher quality evidence and help establish the superiority or non‐inferiority of specific surgical techniques.

## 5 CONCLUSIONS

The study suggests that the improved uniportal thoracoscopic bullectomy with parietal pleurectomy technique shows satisfactory perioperative results. The technical steps and actions implemented in this study were found to be feasible, safe, and potentially effective in reducing recurrence rates of PSP. To validate these results and ascertain the long‐term effects and comparative effectiveness of this method, additional research, such as larger scale studies and randomized controlled trials, is required.

## AUTHOR CONTRIBUTIONS

**Ningning Kang:** Guarantor of integrity of the entire study; study concepts; study design; definition of intellectual content; clinical studies; manuscript preparation. **Hao Zheng:** Definition of intellectual content; literature research; clinical studies; manuscript preparation. **Wei Ge:** data analysis. **Jin‐Xiu Hu:** Statistical analysis. **Wen Liu:** Clinical studies; data analysis; manuscript editing. **Ren‐Quan Zhang:** Guarantor of integrity of the entire study; manuscript review. All authors read and approved the final manuscript.

## CONFLICT OF INTEREST STATEMENT

The authors declare that there is no conflict of interest.

## ETHICS STATEMENT

This retrospective study was approved by the ethics committee of the First Affiliated Hospital of Anhui Medical University, Hefei, China (Approval number: Quick PJ 2023‐01‐21). Because this was a retrospective study, oral consents from the patients were obtained. The written consents were waived by the ethical committee of the First Affiliated Hospital of Anhui Medical University, Hefei, China. All methods were performed in accordance with the relevant guidelines and regulations.

## Data Availability

The data that support the findings of this study are available from the corresponding author upon reasonable request.
